# Gender Impacted Gut Microbiota and Growth Performance in the Blotched Snakehead (*Channa maculata*)

**DOI:** 10.3390/microorganisms12050871

**Published:** 2024-04-26

**Authors:** Chang Fang, Fang Zeng, Shijun Chen, Shuisheng Li, Yuting Yang, Wanjing Lin, Yun Liu, Cheng Peng, Huirong Yang

**Affiliations:** 1 College of Marine Sciences, South China Agricultural University, Guangzhou 510642, China; chauncyfangcms@stu.scau.edu.cn (C.F.); fangzeng@scau.edu.cn (F.Z.); csjscaucms@scau.edu.cn (S.C.); yyt19990518@163.com (Y.Y.); wjl05@stu.scau.edu.cn (W.L.); 2 Zhongshan Innovation Center, South China Agricultural University, Zhongshan 528400, China; 3 Laboratory for Aquatic Economic Animals, Guangdong Provincial Engineering Technology Research Center for Healthy Breeding of Important Economic Fish, Sun Yat-Sen University, Guangzhou 510275, China; lshuish@mail.sysu.edu.cn (S.L.); liuyun703@126.com (Y.L.); 4 Guangdong Key Laboratory of Animal Conservation and Resource Utilization, Institute of Zoology, Guangdong Academy of Sciences, Guangzhou 510260, China; 5 Guangdong Public Laboratory of Wild Animal Conservation and Utilization, Institute of Zoology, Guangdong Academy of Sciences, Guangzhou 510260, China

**Keywords:** blotched snakehead, gender, gut microbiota, grow performance

## Abstract

The blotched snakehead *Channa maculata* is an important economical freshwater species in East Asia. However, there has been relatively little research conducted on the correlation between gender and gut microbes. In this study, 36 of 1000 blotched snakeheads were randomly selected for growth performance measurement and gut microbiota high-throughput sequencing. Results showed that microbial diversity, composition, and metabolic functions were altered by gender and growth performance except the microbial network. In our study, Proteobacteria were the most abundant phylum, with Fusobacteria showing enrichment in males and Bacteroidetes in females. Notably, phylum Deinococcus-Thermus was identified as a significant biomarker. The Cetobacterium was the most abundant genus-level taxon. Furthermore, gut microbes specializing in the production of gut-healthy substances, such as coenzymes and vitamins, were identified as biomarkers in the fast-growing group. Our investigation highlighted the impact of gender on the composition and abundance of gut microbial biomarkers in both males and females, thereby influencing differential growth performance through the modulation of specific metabolic functions.

## 1. Introduction

The blotched snakehead *Channa maculata* is an important economical freshwater species in East Asia, which originated from southern China and Vietnam [1]. It is a type of freshwater fish with high-quality flesh, fewer muscle spines, and rich nutritional value, of whose protein content of fillets could even reach 18~22%. This makes it a valuable source of premium fish products that align with today’s consumer demand for high-quality protein. Meanwhile, this fish has become the most important aquaculture species due to its fast growth rate, strong hypoxia tolerance, ease of intake of artificial compound feed, resistance to transportation, and ease of processing [2,3,4]. Commercial aquaculture systems are expanding rapidly in response to increased market demand for aquatic products. In China, the total annual output of blotched snakehead has reached up to 500,000 tons in 2022 according to the statistics of the authorities [5].

As with other fish species, blotched snakehead exhibits clear sexual dimorphism [6]. The growth rate and body size are both of the most explicit indicators among all sexual dimorphisms, and males of blotched snakehead grow faster than females [7]. Sex-specific differences in growth rates and body size could vary among fish species, highlighting the economic importance of same-sex stock production for aquaculture [8,9]. Field surveys and aquaculture studies have shown that male blotched snakehead exhibited significantly larger body size and growth performance compared to females [10]. On top of other factors such as metabolic levels and hormones, gut microbes are one of the important players in determining nutritional capacity, and gender has a continuous effect on the formation and development of the gut microbiota.

Gut microbiota were regarded as the “Second Genome” in animals and played a key role in regulating growth and development. The growth rate of fish depended on their gut’s ability to digest and absorb nutrients, and the gut microbiota’s ability to metabolize and recycle nutrients [11]. Li and colleagues investigated differences in richness and network structure functions of intestinal microbiota between male and female yellow drums (*Nibea albiflora*). At the phylum level, their intestinal microbiota showed similar composition, while significant differences in the relative abundance of Bacteroidetes, Firmicutes, and Proteobacteria in their intestinal microbiota caused by gender were present [12]. Gruneck et al. revealed that gender had a significant effect on the composition of the gut microbiota during the development of Siamese fighting fish (*Betta splendens*) [13]. An experiment conducted by Navarro-Barrónet demonstrated that feeding male and female zebrafish a high-fat diet results in sex-specific changes in the diversity and composition of their intestinal microbiota [14]. Li and colleagues identified significant distinctions regarding intestinal microbiota in wild largemouth bronze gudgeon (*Coreius guichenoti*) between the male and female. Prior research has demonstrated that the influence of gender on the gut microbiota of the pufferfish Takifugu obscurus manifests primarily in the relative abundance of specific bacterial taxa rather than in the overall community composition. This implies that distinct bacterial communities, particularly those represented by genera like Sphingomonas, may have the potential to impact individual growth rates differently across genders [15]. Therefore, exploring and identifying the gut microbiota in fish individuals based on their sex and body type were crucial for a comprehensive understanding of the intestinal microbial community and its potential applications. The dominant phylum was Proteobacteria, accounting for 97.6%, in males and Tenericutes and Proteobacteria, accounting for 52.3% and 40.5%, respectively, in females [16]. Thus, the gut microbiota played a crucial role in nutrition, immunity, and development, shaping a complex micro-ecosystem within the intestine [15]. Gender has a persistent impact on the formation and development of gut microbiota, even when considering other factors.

Currently, studies on blotched snakehead have generally focused on growth and development, disease, muscle quality, breeding, and sex reversal. But, there has been relatively little research conducted on the correlation between sexual dimorphism and gut microbes. Given the industrial and economic importance of male blotched snakeheads to modern freshwater aquaculture, it is imperative that the gut microbiota, which promote their growth, be investigated. Previous research indicated that genetic development and breeding studies of blotched snakeheads are a “hot issue” for the enhancement in genetic traits and for their use as parents for hybrid snakeheads. Apparently, studies on how gut microbiota impact growth performance in blotched snakehead were still lacking. In this study, we aimed to (i) explore the differences of intestinal microbial community and diversity, (ii) identify the potential microbial taxa that promote growth, and (iii) investigate how gender shapes the discrepancy in the functional composition of gut microbiota.

## 2. Materials and Methods

### 2.1. Experimental Fish and Sample Collection

The experimental blotched snakeheads were obtained from a fish farm in Foshan City, Guangdong Province, China. The blotched snakeheads had an initial body weight of 5.0 ± 0.5 g and were fed on a commercial diet (dry matter: crude protein—48.1%, crude lipids—11.3%, ash—12.3%, carbohydrates—20.1%, gross energy—19.3 kJ/g, and protein/energy ratio—26.0 mg/kJ; Alpha Feed Co., Ltd., Shenzhen, China) twice daily at 9:00 and 15:00 h [17]. The water temperature was at 25~28 °C, pH was 7.0~7.5, and the dissolved oxygen content in water was kept above 5 mg·L^−1^. We employed skilled workers from aquaculture farms to help us with the individual selection, and only adult fish were allowed to be included in our experiments. At 120 days of the culture, 1000 fishes were randomly collected from the farm for growth characterization (including body weight, length, and height) and gender identification. By using a previously developed protocol reported by Han et al. [18], a simple PCR-based genetic sex identification method in the blotched snakehead (Channa maculata) developed by high-throughput sequencing, we identified male (XY) and female (XX) individuals and removed supermale individuals (YY). After ranking the experimental fish on the basis of body weight, the top-ranked nine individuals and the bottom-ranked nine individuals were selected in both male and female groups. The fishes with the maximum weight and minimum weight of both genders were considered as the fast-growth group and low-growth group, respectively. Four groups separated by gender and weight were established as follows: F-F (female fishes with fast growth, *n* = 9), F-S (female fishes with slow growth, *n* = 9), M-F (male fishes with fast growth, *n* = 9), and M-S (male fishes with slow growth, *n* = 9). For the purpose of collecting intestinal content samples, a total of 36 fish contributing to the four groups were individually euthanized in pH-buffered tricaine methanesulfonate (250 mg/L) (Dr. Ehrenstorfer, Augsburg, Germany). The entire intestine was surgically excised under sterile conditions and the contents was collected carefully; we thoroughly mixed the intestinal contents originating from the same group of 3 individuals and obtained 1 sample, and a total of 12 samples were quenched by liquid nitrogen immediately and stored at −80 °C for further treatment. All studies were rigorously conducted in alignment with the South China Agricultural University’s Guide for the Care and Use of Laboratory Animals, and approved by the ethics committee of Laboratory Animal Center of South China Agricultural University (Approval Code: 201805021).

### 2.2. High-Throughput Sequencing

Microbial genomic DNA of the gut microbiota were extracted using commercial kit MagPure Stool DNA KF Kit B (Guangzhou Magen Biotechnology Co., Ltd., Guangzhou, China) according to the manufacturers’ instructions. The quality and concentration of DNA were assessed using a NanoDrop2000 spectrophotometer (Thermo Fisher Scientific, Waltham, MA, USA).

The 16S rRNA V3-V4 regions were amplified using the primers 341F (5′-CCTACGGGNGGCWGCAG-3′) and 806R (5′-GGACTACHVGGGTATCTAAT-3′) [19]. The PCR reactions were conducted using the following program: denaturation at 94 °C for 2 min, 30 cycles of 10 s at 98 °C, then 30 s for annealing at 55 °C and 30 s for elongation at 68 °C, and final extension at 68 °C for 5 min. Purified PCR products were sequenced on an Illumina Nova6000 platform with the PE250 mode in GeneDenovo Biological Technology Co., Ltd. (Guangzhou, China).

### 2.3. Bioinformatic and Statistical Analysis

The raw reads were filtered, assembled, and filtered to obtain clean tags. Then, the clean tags were clustered into operational taxonomic units (OTUs) of more than 97% similarity using the UPARSE (version 9.2.64) pipeline [20]. Classification was determined using the abundant sequences against the GreenGenes database (version 13.8). Alpha diversity indices including Shannon diversity, ACE richness, Pielou evenness, and Faith PD were estimated using the R project. Beta diversities assessing similarity between groups were displayed using non-metric multidimensional scaling (NMDS). OTUs whose abundance > 0 and total proportion > 0.1% were selected for an indicator analysis using the labdsv package in R. The linear discriminant analysis effect size (LefSe) was observed to identify the biomarkers with an LDA threshold value = 2.5. A Venn diagram was used to display the shared and unique OTUs among the groups. The phylogenetic investigation of communities by the reconstruction of unobserved states (PICRUSt) based on the Kyoto Encyclopedia of Genes and Genomes (KEGG) databases and Functional Annotation of Prokaryotic Taxa (FAPROTAX) was employed to predict the function [21]. A principal co-ordinate analysis (PCoA) with a permutation multivariate analysis of variance (Adonis) was applied to describe beta diversity of predicted functions. Differential predicted functions between the fast-growth group (F) and the slow-growth group (S) were analyzed by the negative binomial distribution using DESeq2 and visualized by a volcano plot. The microbial co-occurrence networks at the OTU level based on Spearman’s correlation were visualized by Gephi to estimate the microbial correlations and identify specific species of high connectivity. The Spearman correlation scores were calculated and only robust (Spearman’s *r* > 0.7 or *r* < −0.7) and statistically significant (*p* < 0.01) correlations were retained [22]. The network topological indexes such as the node number, edge number, edge density, degree centralization, betweenness centralization, average clustering coefficient, complexity, and modularity were calculated to describe network properties.

Data of growth performance and network property indices were presented as the mean ± standard error (SE). A two-way analysis of variance (ANOVA) with the Fisher LSD precision test was employed to indicate the differences of microbial diversity caused by growth and gender. Differences with a *p*-value < 0.05 were considered statistically significant.

## 3. Results

### 3.1. Growth Performance Analysis

After the 120-day trial, the total length, height, standard length, and weight of blotched snakeheads showed significant differences among the four groups (Table 1). The two-way ANOVA indicated that the mixed effects of growth performance and sex were not significant, while gender showed remarkable impact on weight (main effect, F = 68.97, *p* = 0.001), full length (main effect, F = 36.155, *p* = 0.001), height (main effect, F = 26.333, *p* = 0.001), and standard length (main effect, F = 46.231, *p* = 0.001).

### 3.2. Microbial Taxonomic Composition

**Figure 1:**
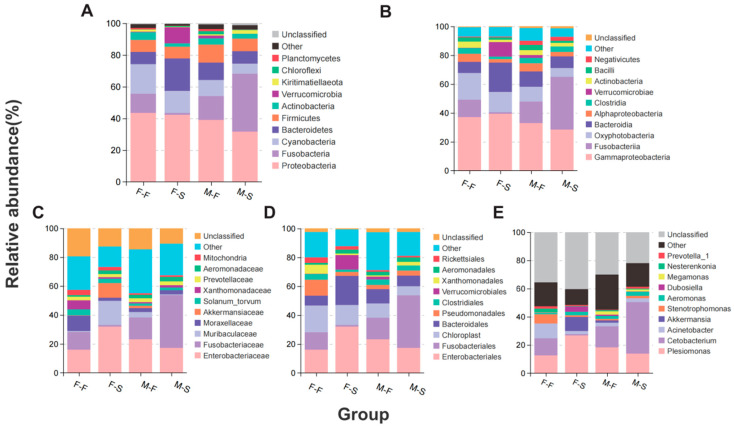
Microbial composition in present study. Histogram of relative abundance at phylum level (**A**), class level (**B**), order (**C**) and family (**D**), and genus level (**E**).

Proteobacteria (31.67~43.35%) were the dominant phylum among the four groups, followed by Fusobacteria (1.10–36.46%), Bacteroidetes (7.93–20.38%), Cyanobacteria (6.32–18.60%), and Fimicutes (7.44–11.14%). The top five phyla cumulatively exceeded 80% for all groups (Figure 1A). The top three genera of each group are described below: (F-F group) *Plesiomonas*, *Cetobacterium*, and *Acinetobacteria*; (F-S group) *Plesiomonas*, *Akkermansia*, and *Dubosiella*; (M-F group) *Plesiomonas*, *Cetobacterium*, and *Aeromonas*; and (M-S group) *Cetobacterium*, *Plesiomonas*, and *Aeromonas*. The abundance of *Cetobacterium* was greater in males than in females. Gammaproteobacteria were the major class across four groups, and Fusobacteria were enriched in male fish (Figure 1B). Order Xanthomonadaceae was only dominant in the group F-F (Figure 1C). Fusobacteriales, Enterobacteriales, and Chloroplasts occupied approximately half of the abundance of all families (Figure 1D). The proportion of Fusobacteria was greater in males (15.00–36.46%) than it was in females (1.10–12.22%) while Bacteroidetes was greater in females (7.64–20.38%) than it was in males (7.93–11.07%). At the genus level, the top ten taxa accounted for approximately 50% (Figure 1E).

### 3.3. Alpha and Beta Diversity of Gut Microbiota

**Figure 2:**
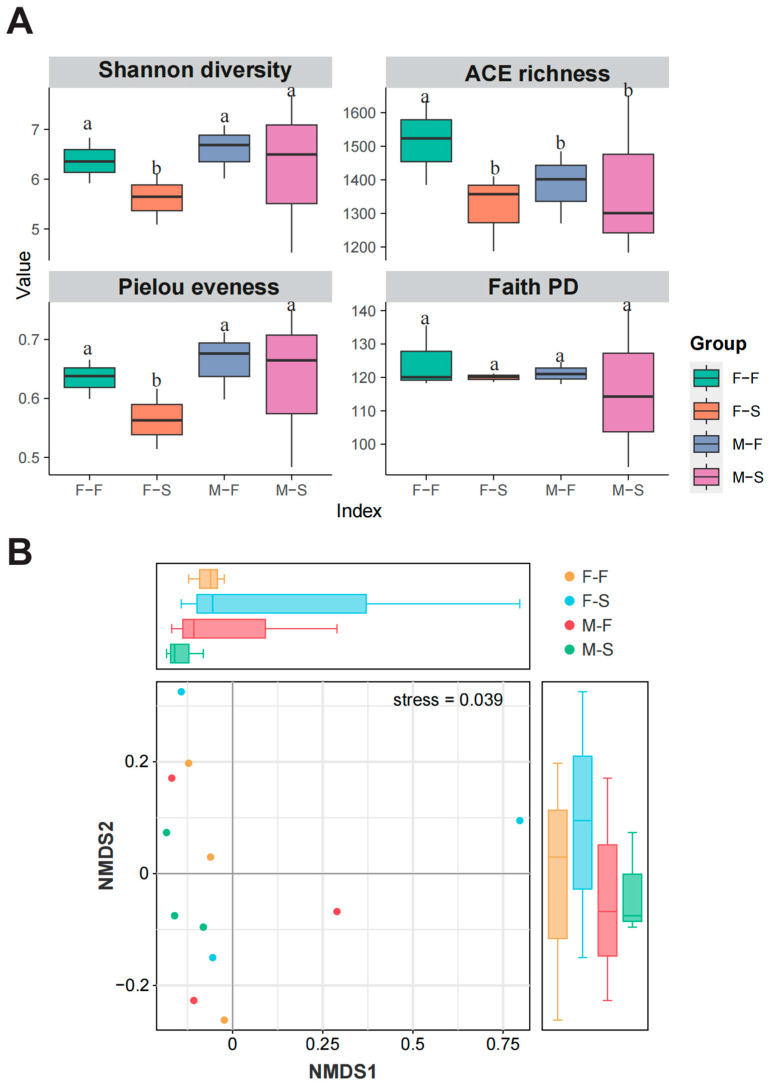
Alpha and beta diversity of microbial community in present study. Alpha diversity, richness, evenness, and phylogenetic diversity are illustrated by boxplot (**A**). NMDS plot shows the distinctions of bacterial structure between groups (**B**). Different lowercase letter labels represent significance between groups. Significant level: *p* < 0.05.

To assess the effect of gender and performance on gut microbiota alpha diversity, Shannon diversity, ACE richness, Pielou evenness, and Faith PD indices were calculated (Figure 2A). All four indices indicated that alpha diversity of the fast-growth group (F, including F-F and M-F) was higher than it was in the slow-growth group (S, including F-S and M-S). The Shannon diversity and Pielou evenness indices were higher in males; nevertheless, the ACE richness was higher in the females. Moreover, the Shannon diversity, ACE richness, and Pielou evenness were significantly higher in F-F than in F-S (*t* test, *p* < 0.05). However, overall, the two-way ANOVA suggested that growth performances (main effect, F = 1.232, *p* = 0.299) and gender (main effect, F = 0.141, *p* = 0.717) in this study both did not have a significant impact on the Shannon diversity of gut microbiota.

The Bray–Curtis distance was applied to calculate the beta diversity using NMDS (Figure 2B). Results showed that separation between the four groups was not significant enough with a stress value of 0.039. Most of the samples were displayed in the negative half of the *X*-axis; moreover, the clusters of F-F and M-S were, respectively, independent despite the cross by other groups. The results indicated that the similarly global composition was shared between the four groups.

### 3.4. Linking Growth Performance and Gender to Biomarkers

**Figure 3:**
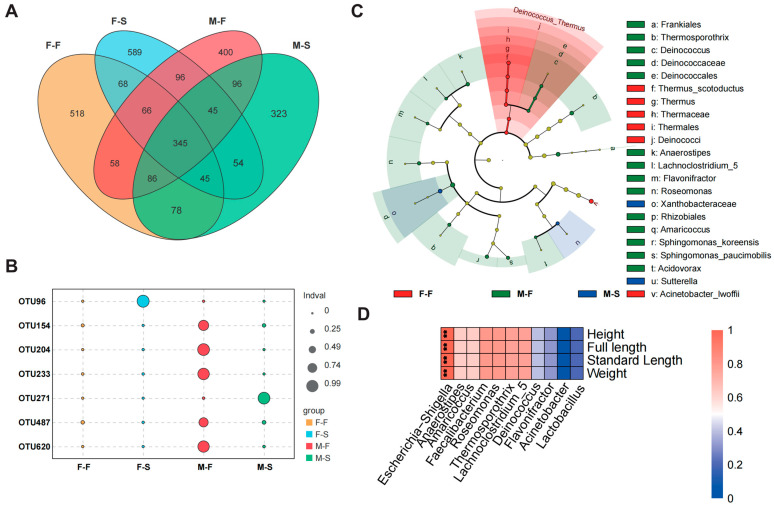
Effect of gender and growth performance on gut microbiota. Venn diagram of OTU distribution (**A**), indicator analysis of keystone potential indicators (**B**), LEfSe analysis revealed the most vital biomarker (**C**), and the genus-level biomarkers correlated to growth performance (**D**). Significant level: **, *p* < 0.01.

The Venn diagram indicates that there were 345 OTUs shared among all groups (Figure 3A). F-F and M-F shared 58 specific OTUs while F-S and M-S shared 54 specific OTUs. Specifically, 518, 589, 400, and 323 OTUs were unique to F-F, F-S, M-F, and M-S, respectively. The all-shared bacterial OTUs were taxonomically assigned into 9 phyla, 16 classes, 30 orders, 46 families, and 61 genera. Proteobacteria was recognized as the most abundant core phylum between groups. Family Enterobacteriaceae from class Gammaproteobacteria was the dominant taxa in the core microbiota. The shared bacterial OTUs between F-F and M-F were classified into 8 phyla, 13 classes, 22 orders, 33 families, and 32 genera. The dominant taxa in the shared microbiota between F-F and M-F was family Burkholderiaceae from class Gammaproteobacteria. A total of 7 phyla, 11 classes, 17 orders, 23 families, and 25 genera were identified in the shared OTUs between F-S and M-S, whose major taxa were the same as the core microbiota among all groups.

The indicator analysis suggested that OTU96 (phylum Firmicutes, genus *Quinella*) and OTU271 (phylum Proteobacteria) with IndVal being more than 0.9 were the most probable indicators in F-S and M-S, respectively (Figure 3B). Most indicators were identified in the M-F. Five OTUs with IndVal > 0.7, including OTU154 (phylum Proteobacteria, genus *Acinetobacter*), OTU204 (phylum Firmicutes, genus *Lactobacillus*), OTU233 (phylum Firmicutes, genus *Faecalibacterium*), OTU487 (phylum Proteobacteria, genus *Escherichia-Shigella*), and OTU620 (phylum Proteobacteria, genus *Roseomonas*), were considered as the strongest potential indicators in M-F. The LEfSe analysis was used to further determine the prominent bacterial taxa driving the distinctions of gut microbiota. Results with a threshold value = 2.5 displayed that the counts of enriched biomarkers in M-F, F-F, and M-S were 14, 6, and 2 (Figure 3C). Obviously, there were more enriched biomarkerable taxa in the fast-growth groups and male groups. Biomarkers of M-F were more widely distributed in phylogenetic evolution. At the genus level, *Anaerostipes* (LDA score = 2.97), *Deinococcus* (LDA score = 2.90), *Amaricoccus* (LDA score = 2.70), *Flavonifractor* (LDA score = 2.53), *Roseomonas* (LDA score = 2.73), *Acidovorax* (LDA score = 2.55), *Thermosporothrix* (LDA score = 3.10), and *Lachnoclostridium_5* (LDA score = 2.51) were the biomarkers identified with M-F. The shared biomarker between F-F and M-F was enriched in phylum Deinococcus-Thermus. At the genus level, *Escherichia-Shigella* significantly showed positive correlation to all four indices (*p* < 0.05, Figure 3D).

### 3.5. Structure of Potential Metabolic Function and Microbial Network

**Figure 4:**
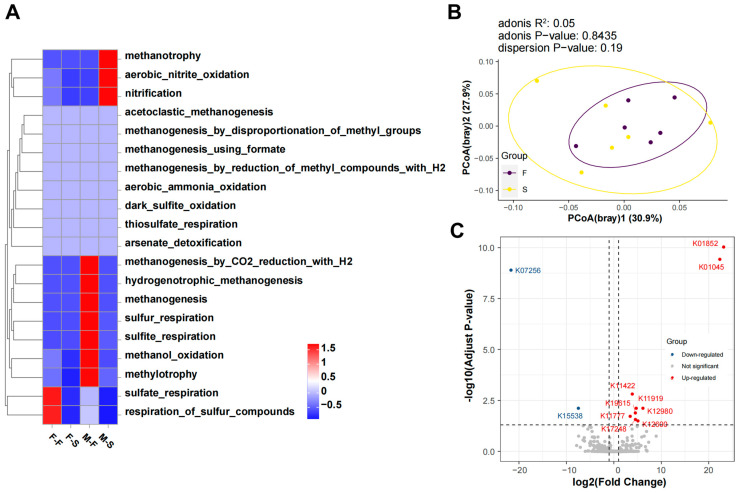
Predicted metabolism of gut microbiota. Correlation between group and microbial functions (**A**). PCoA analysis indicated beta diversity of microbial functions (**B**). Key KOs are illustrated by volcano plot with threshold value = 1 (**C**).

The FAPROTAX analysis showed the metabolic distinctions between groups. A heatmap indicated that the respiration of sulfate and sulfur compounds was the main microbial function in F-F (Figure 4A). However, several kinds of methane and sulfur metabolisms, including methanogenesis by CO_2_ reduction with H_2_, hydrogenotrophic methanogenesis, methanogenesis, sulfur respiration, sulfite respiration, methanol oxidation, and methylotrophy, were more greatly enriched in M-F. Methanotrophy, aerobic nitrite oxidation, and nitrification were the dominant microbial metabolisms with M-S. To further demonstrate the structural variation of microbial metabolism, the principal co-ordinate analysis (PCoA) based on Bray–Curtis distance was employed. The two axes accounted for approximately 60% of the result of PCoA (Figure 4B). Results of Adonis showed that there was not a significant difference in microbial functions within group F and group S, while the structure of group F was more contracted and centralized than that of group S.

In order to gain a thorough understanding of up- and down-regulated metabolism, a differential expression analysis was performed to identify the keystone orthologies (threshold: log_2_(Fold Change) = 3.5, *p*-value = 0.05) (Figure 4C). Results showed that 9 orthologies were significantly up-regulated, including K01852 (lanosterol synthase), K01045 (arylesterase or paraoxonase), K119119 (type VI secretion system lysozyme-related protein), K11422 (N-trimethyltransferase), K19615 (insecticidal toxin), K12980 (flagellar motor switch protein FliG), K11777 (HAD superfamily phosphatase), K12600 (superkiller protein 3), and K17248 (acetylgalactosaminyltransferase). Only K07256 (taurine dehydrogenase large subunit) and K15538 (glycoprotein endo-alpha-1,2-mannosidase) were down-regulated.

**Figure 5:**
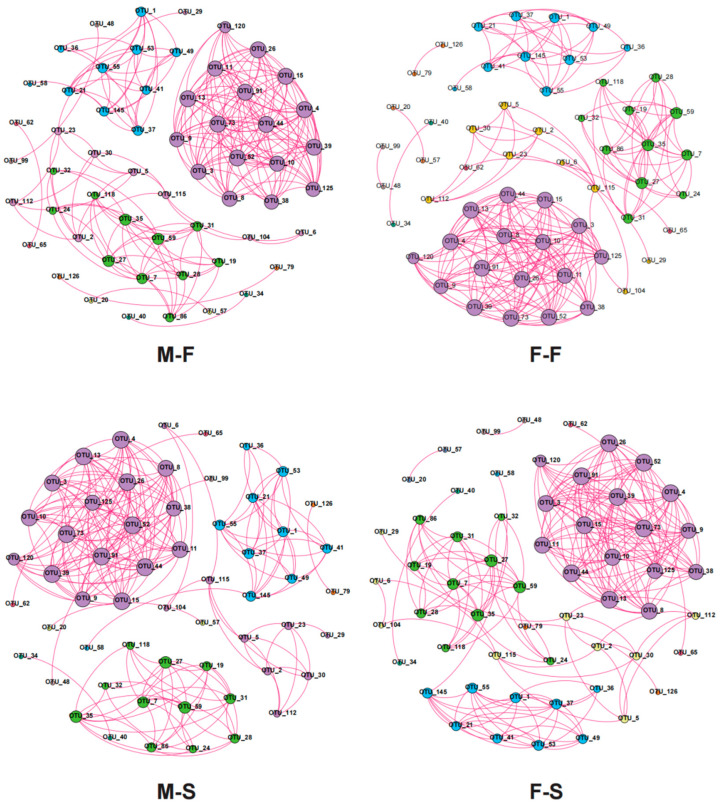
Co-occurrence network at OTU level for each group.

The microbial network was created with a Spearman correlation analysis with *p* < 0.05 and correlation > 0.7 (Figure 5). The four stable and significant co-occurrence networks had positive correlation. Profiles and parameters of the networks indicated no differences between groups (Table 2). The mean parameters of each network are described as follows: nodes = 56, edges = 218, edge density = 0.14, degree centralization = 0.14, betweenness centralization = 0.01, average clustering coefficient = 0.93, complexity = 3.90, modularity = 0.62.

## 4. Discussion

Previous studies on blotched snakeheads have focused on its variance of meat quality and nutritional value caused by gender, whereas there is not enough information on the intestinal microbiota. Gut microbiota played a vital role to the host, especially the fish, and understanding the regulation and composition of the gut microbiome in blotched snakeheads was crucial in comprehending their viability and growth capacity in cultured environments [23]. This study aimed to determine the change in microbial community composition, diversity, predicted functions, and co-occurrence networks grouped by gender and growth performance.

Proteobacteria were the most abundant core phylum between groups and are also recognized as a common type of bacteria found in natural habitats. This diverse group of bacteria includes various genera and species, causing distribution in fish to vary depending on the specific type, which was distributed throughout the aquatic environment. Chang et al. found that after the infection of *Aeromonas hydrophila*, the abundance of Fusobacteria increased [24], despite that it was considered as common taxa found in other studies of freshwater fishes and environments [25,26]. Various investigations have concluded that Bacteroidetes can break down complex polymers, leading to easier food digestion and nutrient absorption, especially for components found in vegetarian meals, which was consistent with the increased crude fiber content added to the diets fed to blotched snakeheads [27]. Moreover, they may also contribute to the host’s growth by assisting in digestion and nutrient acquisition [28,29]. This suggested that differences in the composition and abundance of the gut flora of males and females may be an important source of differences in their growth.

At the genus level, *Cetobacterium* was the most abundant taxa. The commensal micro-aerotolerant anaerobic bacteria *Cetobacterium somerae* played a significant ecological role in the intestinal tracts of freshwater fish [30]. Notably, the metabolites of *Cetobacterium* were highly concentrated in vitamin B12, which was believed to enhance the overall health of fish [31]. The results of the alpha diversity analysis revealed that the microbial communities in males exhibited higher diversity and evenness, whereas those in females had more richness. This suggests that females may harbor a greater variety of microorganisms with unique functions and occupying different ecological niches within their gut.

Microbial networks are intricate structures that denote the interactions between diverse microorganisms in a community [32]. These interactions can involve various relationships such as symbiotic, competitive, and coexistent, shaping the ecosystem of the microbial community. Microbial networks were distinctive, given that microbial communities fluctuate across environments and ecosystems [33]. Nevertheless, they were also uniform, as some ecological principles and patterns were commonly observed within microbial networks. In the present study, although there were marked differences in microbial community structure, taxonomy composition, and functional distributions among groups, the topological networks remained similar. As a result, there was convergence in the microbial networks of the four sub-groups differentiated by growth and sex. This further implies that although the microbial community was highly variable among individuals, the symbiotic and competitive relationships of the microorganisms were quite similar among individuals of the same species [34].

Some microorganisms played a central role in the microbial community, performing key functions and producing metabolites that promoted the host’s growth performance. The Red Queen Hypothesis proposed an objective explanation for species evolution, focusing on the need for certain species to constantly evolve in order to ensure their survival in the field of biology [35]. To maintain their niche in the ecosystem, species evolved to adapt to and counteract the evolution of other species. This view emphasized the importance of ecological competition and asserts that organisms must evolve to adapt to the evolution of other species in order to maintain their position in the ecosystem. It is known to us that females have a lower growth rate compared to males in nature and, as a result, the ecological niche and the nutritional conditions in the female gut were less favorable for the microorganisms. Therefore, the taxonomic and evolutionary developmental status of gut biomarkers at the OTU level was extensive in males. However, it was limited and sparse in females. This could be an explanation for the higher amount of biomarkers in males and the lower amount of biomarkers in females.

Family Burkholderiaceae, the dominant taxa shared by F-F and M-F, were regarded as hazardous microorganisms that contain extreme virulences capable of disrupting immune function [36]. It was amazing that some members of Deinococcus-Thermus, which was identified as a biomarker in F-F and M-F, exhibited remarkable resistance to harsh environmental conditions, like oxidative stress and high toxics [37,38]. This evidence suggested that non-numerically dominant biomarkers confer significant functional advantages in specific conditions and regulate the health of the host’s gut microbiota through pathways that include metabolites and community sensing. Significant biomarkers at the genus level could explain the differences between fast growth and slow growth, and could further be the composition of potential comprehensive prebiotic formulations. Genera with metabolic functions of short-chain fatty acids (SCFAs) could be a potential microbial contribution to fast growth. For instance, Roseomonas was capable of producing SCFA, while certain strains of Anaerostipes have the ability to metabolize dietary inositol into short-chain fatty acids such as propionate and butyrate [39,40]. Also, the increase in populations of Faecalibacterium with a concomitant promotion in butyrate production was observed [41]. Acinetobacter can be a key microbial genus for improving gut microbiota dysbiosis in high-sucrose diets (HCDs) by activating intestinal pathogenic bacteria in response to inflammation [42]. Therefore, more research is still needed to determine the key microorganism of blotched snakeheads in growth.

By creating a viable environment for survival, regulating immune responses, and providing essential nutrients for beneficial microorganisms to thrive while suppressing harmful microorganisms, the host maintained the balance of microorganisms in the gut [43]. The health of the host depended on this balance of host–microbial interactions. Meanwhile, gut microbiota had a broad-spectrum influence on the host, encompassing diverse aspects of digestion, immunity, and the nervous system [34]. They assisted in the digestion and absorption of nutrients, preservation of the intestinal mucosal barrier, modulation of the immune response, and synthesis of vitamins and other bioactives, and ultimately impacted metabolism and body weight [44,45]. In our study, Escherichia-Shigella, which was considered as a pathogen, showed positive correlation with four indices of growth performances. We noticed that Escherichia-Shigella was not very abundant in any group, so it may be that the metabolic functions of other microorganisms weakened its pathogenicity and abundance. In our study, Escherichia-Shigella, which was considered as a pathogen, showed positive correlation with four indices of growth performances. We noticed that Escherichia-Shigella was not very abundant in any group, so it may be that the metabolic functions of other microorganisms weakened its pathogenicity and abundance. Escherichia-Shigella was commonly found in gut samples. These species pose a threat to fish health in a physiologically unbalanced environment [46], while in the conditions of the current study, their relative abundance probably did not affect the fish to the point of developing disease. It was obvious that sulfur-related metabolism and methanogenesis were the dominant microbial function of M-F and F-F, while methanotrophy and nitrogen-related metabolism occupied the larger proportion in F-S and M-S. Sulfur cycling is critical in microbial biochemistry for the synthesis of coenzymes, including coenzyme A and coenzyme M; the utilization of sulfur compounds such as thiols; and the production of vitamins such as thiokinase and thione [47]. In cellular metabolism, coenzyme A and coenzyme M perform critical catalytic and electron transfer functions [48]. By directly or indirectly participating in the sulfur cycle, microorganisms influence the synthesis of these coenzymes and regulate the energy metabolism of organisms [49]. It played an integral role in supporting bacterial metabolism and overall microbial community dynamics. The microbial nitrogen cycle influenced and regulated the nutrition of individual organisms through the transformation of nitrogen into a variety of forms in which microorganisms play a critical role. The nitrogen cycle regulated the form and availability of nitrogen in organisms, directly or indirectly affecting protein and nucleic acid synthesis [50]. Ultimately, the nitrogen cycle had a significant impact on the growth and development of organisms. It is worth noting that the differences in microbial function within groups F and S were still not deemed significant; the structures within group F were more contracted and centralized than those within group S. It was evident that, besides differences triggered by gender, preferences in gut microbial function can also contribute to variations in growth performance. In fast-growing populations, gut microbes primarily produced gut-healthy substances such as coenzymes and vitamins, whereas in slow-growing populations, metabolizing as well as digesting and absorbing substances took precedence. It was suggested that the screening of microorganisms that are able to secrete small molecules, as well as important organic compounds such as coenzymes and vitamins, from individuals with growth advantages has significant potential for research and practical applications [51,52].

## 5. Conclusions

To summarize, we found that microbial diversity, composition, and metabolic functions were influenced by both gender and growth performance, but not the microbial network. Specifically, male blotched snakeheads exhibited higher Shannon diversity and Pielou evenness indices, while females showed higher ACE richness. The most abundant phylum was Proteobacteria, with Fusobacteria enriched in males and Bacteroidetes enriched in females. *Cetobacterium* was the most abundant genus. Phylum Deinococcus-Thermus emerged as a significant biomarker. In fast-growing groups, gut microbes were likely to produce gut-healthy substances, such as coenzymes and vitamins, due to the prevalence of sulfur metabolism. Our findings suggested that gender influences the composition and abundance of gut microbial biomarkers, leading to differential growth performance by shaping specific metabolic functions. Further research is needed to understand the underlying causes of these growth differences. Overall, this study offered new insights for understanding and exploiting the potential of the gut microbiome.

## Figures and Tables

**Table 1:** The basis growth performance parameters of each group.

	Group (Mean ± SE)			
	F-F (*n* = 9)	F-S (*n* = 9)	M-F (*n* = 9)	M-S (*n* = 9)
Total length (cm)	32.33 ± 0.76 ^a^	24.30 ± 0.62 ^b^	34.93 ± 1.01 ^a^	27.40 ± 4.26 ^b^
Height (cm)	6.43 ± 0.06 ^a^	5.13 ± 0.42 ^b^	6.70 ± 0.44 ^a^	5.37 ± 0.65 ^b^
Standard length (cm)	27.90 ± 1.15 ^a^	20.90 ± 0.56 ^b^	30.70 ± 0.75 ^a^	23.53 ± 3.29 ^b^
Weight (g)	424.07 ± 38.13 ^a^	184.97 ± 25.02 ^b^	540.43 ± 50.34 ^a^	262.50 ± 83.75 ^b^

F-F: Female—fast; F-S: Female—slow; M-F: Male—fast; M-S: Male—slow. Different lowercase letter labels represent significance between groups. Significant level: *p* < 0.05.

**Table 2:** The topographic parameters of co-occurrence networks.

	Group (Mean ± SE)				F	*p*
	F-F (*n* = 3)	F-S (*n* = 3)	M-F (*n* = 3)	M-S (*n* = 3)		
Nodes	56.67 ± 0.58	56.00 ± 1.73	56.00 ± 1.00	55.33 ± 2.89	0.281	0.838
Edges	220.67 ± 5.77	211.00 ± 22.52	219.00 ± 7.81	222.67 ± 2.31	0.517	0.682
Edge density	0.14 ± 0.00	0.14 ± 0.01	0.14 ± 0.01	0.15 ± 0.01	1.036	0.427
Degree centralization	0.14 ± 0.01	0.14 ± 0.01	0.14 ± 0.01	0.15 ± 0.00	0.488	0.700
Betweenness centralization	0.01 ± 0.00	0.01 ± 0.00	0.01 ± 0.00	0.01 ± 0.00	1.484	0.291
Average clustering coefficient	0.93 ± 0.01	0.92 ± 0.00	0.93 ± 0.00	0.93 ± 0.00	1.169	0.380
Complexity	3.89 ± 0.06	3.76 ± 0.29	3.91 ± 0.15	4.03 ± 0.17	1.018	0.434
Modularity	0.62 ± 0.01	0.62 ± 0.02	0.62 ± 0.01	0.61 ± 0.01	0.690	0.583

F-F: Female—fast; F-S: Female—slow; M-F: Male—fast; M-S: Male—slow.

## Data Availability

Data are contained within the article.
